# Anti-Inflammatory and Anti-Remodelling Effects of ISU201, a Modified Form of the Extracellular Domain of Human BST2, in Experimental Models of Asthma: Association with Inhibition of Histone Acetylation

**DOI:** 10.1371/journal.pone.0090436

**Published:** 2014-03-03

**Authors:** Cristan Herbert, Alexander M. Shadie, Melissa M. Bunting, Nicodemus Tedla, Linda Garthwaite, Araluen Freeman, Hyouna Yoo, Sang-Ho Park, Rakesh K. Kumar

**Affiliations:** 1 Inflammation and Infection Research Centre, School of Medical Sciences, University of New South Wales, Sydney, Australia; 2 Isu Abxis Co., Ltd., Seoul, Korea; French National Centre for Scientific Research, France

## Abstract

There are few alternatives to glucocorticosteroids for treatment of asthma. We assessed the activity of a novel protein drug designated ISU201, the extracellular domain of the human cell surface protein BST2, stabilised by fusion with the Fc region of IgG, in mouse models of mild chronic asthma and an acute exacerbation of asthma. The ability of ISU201 to suppress airway inflammation and remodelling was compared with that of dexamethasone. Female BALB/c mice were systemically sensitised with ovalbumin, then received controlled low-level challenge with aerosolised ovalbumin for 6 weeks, which induced lesions of mild chronic asthma, and were treated with drugs during the final 2 weeks. Alternatively, sensitised mice received 4 weeks of chronic low-level challenge and were treated 24 and 2 hours before a final single moderate-level challenge, which triggered acute airway inflammation simulating an asthmatic exacerbation. Inflammation and remodelling were quantified, as was the expression of pro-inflammatory cytokines in bronchoalveolar lavage fluid and tissues. To identify cellular targets of ISU201, we assessed the effects of the drug on activated lymphocytes, macrophages and airway epithelial cells. In the model of mild chronic asthma, ISU201 was as effective as dexamethasone in suppressing airway inflammation and most changes of remodelling. In the model of an allergen-induced acute exacerbation of chronic asthma, ISU201 was also an effective anti-inflammatory agent, although it was less active than dexamethasone. The drug acted on multiple cellular targets, suppressing production of pro-inflammatory cytokines by lymphocytes and macrophages. ISU201 significantly reduced acetylation of histone H4 in airway epithelial cells, suggesting at least one potential mechanism of action. We conclude that in these models of asthma, ISU201 is a broad-spectrum inhibitor of both airway inflammation and remodelling. Thus, unlike drugs which target specific mediators, it could potentially be an alternative or an adjunct to glucocorticoids for the treatment of asthma.

## Introduction

Asthma is one of the most common chronic diseases affecting children, especially in economically developed nations. For example, in Australia the prevalence of doctor-diagnosed asthma is ≈10% across all ages and ≈16% in children aged 8–9 years [1]. Clinically, the illness is typified by episodic breathlessness and wheezing, together with hyper-responsiveness of the airways to a variety of stimuli. Underlying these manifestations is chronic inflammation of the conducting airways and a variety of structural changes collectively referred to as airway remodelling [2].

Most asthma of childhood onset and a significant proportion of asthma of later onset is allergic, characterised by accumulation in the airway mucosa of activated CD4^+^ T-lymphocytes with a Th2 pattern of cytokine secretion i.e. predominantly interleukin (IL) -4, IL-5 and IL-13; mast cells and macrophages, notably within the airway epithelium; and especially during an acute attack, recruitment of numerous eosinophils [2], [3]. The ongoing airway inflammation and remodelling may eventually be associated with the development of airflow obstruction which is either not reversible or only partially reversible by short-acting β_2_-agonists [4].

Most of the morbidity and healthcare costs of asthma are a consequence of acute exacerbations, which may be triggered by high level exposure to allergen but are more often related to superimposed viral infections, especially by rhinoviruses [5], [6]. In this setting, there is not only inflammation in response to the viral infection but also an exaggerated pattern of allergic inflammation of the airways, reflecting the interaction between innate host defence responses and adaptive immunity [7], [8].

Inhaled glucocorticosteroids are the mainstay of therapy for asthma, because of their ability to suppress allergic inflammation in most patients with mild to moderate disease. Especially in combination with long-acting β_2_-agonists, glucocorticoids effectively control the clinical manifestations of asthma [9]. However, corticosteroid therapy may be less useful for controlling airway remodelling [10]. A proportion of patients with acute exacerbations of their asthma are relatively steroid-resistant [11]. Currently, few therapeutic alternatives to glucocorticoids are available, especially for acute exacerbations of asthma.

Appropriate assessment of the potential of novel anti-inflammatory agents requires realistic pre-clinical models which simulate the chronic airway inflammation and remodelling of ongoing asthma, as well as the acute inflammation of an exacerbation. We have described a mouse model of asthma that involves long-term challenge of sensitised mice with carefully controlled low mass concentrations of aerosolised ovalbumin (OVA) (≈100–1000 times lower than used in conventional models) [12]. The model exhibits changes of mild chronic asthma that closely resemble the human disease, both in terms of pattern and spatial distribution of cellular responses, and has been widely acknowledged to represent a significant improvement in terms of the fidelity with which it reproduces features of human asthma [13], [14], [15]. We have also established a model of an allergen-induced acute exacerbation of chronic asthma, in which following low-level challenge for 4 weeks, animals are briefly exposed to a single moderate-level challenge with allergen. This is associated with more marked airway inflammation, as well as a pattern of airway hyper-responsiveness distinct from that seen in the chronic challenge model, reflecting the distal airway involvement [16]. In the latter model, we have shown that activation of CD4+ T-lymphocytes during an acute exacerbation may be driven by activated alveolar macrophages (AM) [17].

In the present study, we assessed the activity of a novel drug known as ISU201 (developed by Isu Abxis Co., Ltd.) in these experimental models. The active moiety of ISU201 is the extracellular domain (ECD) of the human cell-surface protein known as bone marrow stromal cell antigen 2 (BST2), which is stabilised for delivery by fusion with the Fc region of IgG [18]. We found that ISU201 was a broad-spectrum inhibitor of airway inflammation and remodelling, which was effective in both the experimental models of mild chronic allergic asthma and an acute exacerbation of asthma. Similarly to treatment with the glucocorticoid dexamethasone, ISU201 suppressed the expression of multiple inflammatory cytokines by both lymphocytes and macrophages, and inhibited macrophage function. Moreover, these effects were associated with suppression of histone acetylation in airway epithelium. These findings were confirmed in further studies in vitro. Thus ISU201 may not have the limitations of therapies targeted at specific mediators, and could potentially be valuable for the treatment of asthma.

## Methods

### Ethics Statement

All experimental procedures were approved by the Animal Care and Ethics Committee of the University of New South Wales (reference numbers: 08/09B and 11/50A).

### Mice, Sensitization and Challenge

**Figure 1:**
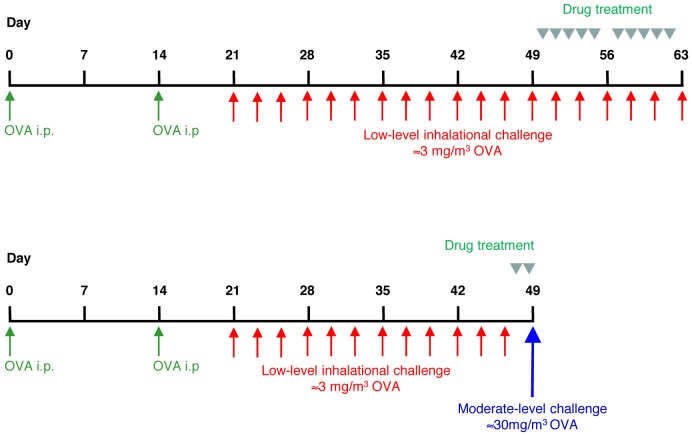
Models of asthma. Timelines for sensitisation, inhalational challenges and drug treatment for the models of chronic asthma and an allergen-induced acute exacerbation of asthma.

The protocols employed have previously been described in detail [12], [16]. Briefly, specific pathogen free female BALB/c mice aged 7–8 weeks (weight 18–20 grams) (Animal Resources Centre, Perth, Western Australia) were systemically sensitised by intraperitoneal injection of 50 µg of alum-precipitated OVA (Grade V, ≥98% pure, Sigma Australia; unless otherwise specified, all chemicals were from this source) 21 and 7 days before inhalational challenge, then exposed to aerosolised OVA in a whole body inhalation exposure chamber (Unifab Corporation, Kalamazoo, MI) [12]. The chronic low-level challenge involved exposure to ≈3 mg/m^3^ aerosolised OVA for 30 minutes/day on 3 days/week for 6 weeks. The acute exacerbation model involved low-level challenge for 4 weeks, followed by a single moderate-level challenge (≈30 mg/m^3^) (Fig. 1). During inhalation exposures, mice were held in flow-through wire cage racks (Unifab Corporation, Kalamazoo, Michigan, USA). Filtered air was drawn through the inhalation chamber (0.5 m^3^) at a flow rate of 250 L/min, and an aerosol of OVA was generated from a 2.5% solution by controlled delivery of compressed air to a sidestream nebuliser (Niche Medical, Australia). Particle concentration within the chamber was continuously monitored using a DustTrak 8520 instrument (TSI, St Paul, MN).

### Drug Treatment

Dexamethasone was administered by gavage (cyclodextrin compound, equivalent to 1 mg/kg; we have previously shown that this dose maximally suppresses inflammation in our model [19]). Recombinant BST2 ECD [18] was fused with the Fc region of IgG via overlap PCR, and a signal sequence of human tissue-type plasminogen activator was engineered on to the 5′ end to facilitate secretion of the protein designated ISU201. This engineered protein is covered by patents in North America, Europe and Australia. ISU201 was administered by intraperitoneal injection (4 or 20 mg/kg: these doses were based on complete dose-response studies conducted by Isu Abxis Co., Ltd. in other models of inflammatory disease). For the chronic challenge experiments, animals were treated on 5 days/week in weeks 5 and 6 of the inhalational exposure period. For the acute exacerbation experiments, doses were administered 24 and 2 hours prior to the single moderate-level challenge. Positive control groups received injections of saline vehicle. Naïve animals were used as negative control groups. Animals were randomly allocated to cages and cages were randomly allocated to experimental groups, which typically comprised 8 animals. Results were replicated in at least 2 separate experiments.

### Assessment of Cell and Tissue Response

#### Bronchoalveolar lavage

Mice were killed by exsanguination following an overdose of sodium pentobarbital at 4 hours after the final airway challenge (this time point was selected on the basis of our earlier studies demonstrating that in the setting of prior chronic challenge, inflammation and cytokine responses develop more rapidly [16]) and lungs were perfused with saline to remove blood from the pulmonary capillary bed. The trachea was cannulated and bronchoalveolar lavage (BAL) performed by inflating the lungs with 3×1 mL of PBS, while gently massaging to maximise cell recovery. Cells were pelleted by centrifugation and BAL fluid was collected and stored at –20°C for immunoassays.

#### Airway inflammation

Changes of inflammation and remodelling were assessed in histological sections of the longitudinally orientated trachea and the mid-zone of the left lung. Intraepithelial eosinophils and nuclear profiles in the lamina propria were counted and expressed as number of cells per 100 µm basement membrane, as previously described [12]. Immunoperoxidase staining for CD3^+^ T-lymphocytes was performed using a rabbit antibody to a peptide of the CD3 ε chain (Sigma). Eosinophils in lung tissue were identified using a cyanide-resistant peroxidase staining technique [20]. Cells were counted at ×40 magnification using Spot imaging software (Diagnostic Instruments, Sterling Heights, MI). Results were expressed as number of cells per mm^2^ of parenchymal tissue. The validity and reliability of the morphometric techniques we employed have been established in previous reports [12], [21].

Alternatively, relative numbers of eosinophils recruited into the lungs were quantified using a colorimetric assay for eosinophil peroxidase (EPO), adapted from previously described methods [22].

#### Airway remodeling

Subepithelial collagenisation and epithelial hypertrophy were assessed in reticulin-stained sections of the trachea as previously described [12] and expressed as the mean thickness for each animal. Goblet cell changes were assessed in periodic acid-Schiff stained lung tissue. The largest visible airway, usually the left main bronchus, was assessed in each section. Positively stained cells were semi-quantitatively graded on a scale from 0–4, where a grade of 0 =  <1% positive cells, 1 = 1–3%, 2 = 4–10%, 3 = 11–30%, and 4 =  ≥31%.

### Cytokine Expression

#### Airway tissue

Proximal airway tissue was isolated by blunt dissection as previously described [23]. In brief, two pairs of forceps were used to tease lung parenchyma from the larger airways, leaving several generations of airway attached to the trachea. RNA was extracted using TriReagent. Samples were treated with DNase (Turbo DNase, Ambion, Scoresby, Australia) and reverse transcribed into cDNA using Superscript III (Invitrogen). Quantitative real time PCR was used to assess expression of cytokines, with detection of amplified products using SYBR green (BioLine, Tauton, MA). Primers were either custom-designed in house or were obtained from Qiagen Australia. Reactions were performed using a LightCyler 480 (Roche Diagnostics, Sydney, Australia) and expression was normalised to hypoxanthine-guanine phosphoribosyl transferase (HPRT).

#### Macrophages

AM were purified from BAL cells by adherence, then re-suspended in TriReagent for extraction of RNA. Adherent cells were >90% AM by morphological criteria and immunostaining for F4/80. For some experiments, AM were cultured in RPMI-1640 under non-adherent conditions in polyethylene tubes (Minisorp, Nunc) for 4 hours with or without ISU201, prior to isolation by adherence. For other experiments, similar studies were performed using MH-S cells, which are derived from BALB/c alveolar macrophages and retain morphological and functional characteristics of highly differentiated macrophages [24], in adherent culture. MH-S cells were activated by prior overnight culture in the presence of 20 ng/mL of IL-33.

#### Peribronchial lymph node (PBLN) cells

Lymph nodes surrounding the trachea and main bronchi were collected from 2–3 animals and pooled to yield 3 samples per treatment group. For flow cytometry of cells from animals treated in vivo, disaggregated cells were used immediately. For assessment of cytokine production following restimulation with antigen and drug treatment in vitro, cells were cultured in 96-well U-bottom microplates in 200 µl of RPMI1640 containing 10% fetal bovine serum and antibiotics, at 0.5×10^6^ cells/well in the presence or absence of 1 mg/mL of OVA [25]. Cells were treated with 1, 3 or 10 µg/mL of ISU201, or 4, 40 or 400 ng/mL of dexamethasone for 72 hours and culture supernatants were collected.

#### Protein immunoassays

The concentration of cytokines in undiluted BAL fluid or in culture supernatants of PBLN cells was measured using a fluorescent multiplex immunoassay (Mouse 23-Plex panel, Biorad Laboratories, Hercules, CA) according to the manufacturer’s instructions.

### Flow Cytometry of PBLN Cells

Expression of surface markers on PBLN cells was assessed using fluorochrome-conjugated antibodies to mouse CD3, CD4, CD8, CD19 and CD25 (BD Pharmingen). Negative controls were cells incubated with the corresponding labelled and isotype-matched immunoglobulins. A 4-channel FACSCalibur flow cytometer (Becton Dickinson, San Jose, CA, USA) was used to acquire fluorescence data.

### Co-incubation of MH-S Macrophages and CD4^+^ T-lymphocytes

CD4^+^ T-lymphocytes were isolated from PBLN cells using a FlowComp magnetic bead isolation kit (Invitrogen, Carlsbad, CA) according to the manufacturer’s protocols. Cell purity was assessed by flow cytometry and was determined to be ≈98.0% CD3^+^/CD4^+^. MH-S cells, activated by IL-33 in the presence or absence of ISU201 or dexamethasone, were co-incubated with CD4^+^ T-lymphocytes at a ratio of 4×10^5^∶1×10^6^ cells/well in 24 well tissue culture plates for 4 hours. Non-adherent CD4^+^ T-lymphocytes were then collected and RNA was extracted.

### Assessment of Histone Acetylation in Airway Epithelium

#### Histone acetylation in airway tissues

Immunoperoxidase staining for acetylated histones H3 and H4 was performed on paraffin sections of trachea after antigen retrieval, using rabbit monoclonal anti-H3(Lys9) and rabbit polyclonal anti-H4(Lys5) (Cell Signaling, Danvers, MA). Positively-stained nuclei were counted in at least 300 airway epithelial cells (AEC) and expressed as a percentage.

#### Histone acetylation in AEC in culture

Primary cultures of AEC from tracheal explants from 8-week-old BALB/c mice were established as previously described [26], [27] and used at passage 6. Cells were stimulated for 18 hours in serum-free medium with 10 µg/mL of polyinosinic:polycytidylic acid (poly I:C, Invivogen), a synthetic analogue of dsRNA which acts as a TLR3 agonist, with or without ISU201 (10 µg/mL) or dexamethasone (40 ng/mL). Proteins were extracted using cell lysis buffer (Cell Signaling) and samples were loaded in duplicate on to 15% acrylamide gels. One gel was blotted on to nitrocellulose and the other was silver stained. The Western blot was probed for acetylated histone H4 using the anti-H4(Lys5) antibody. Detection of bound antibody was by chemiluminescence using the Western Lightning-ECL substrate (Perkin Elmer), which was visualised with an ImageQuant LAS 4000 (GE Healthcare). Densitometry was performed using ImageJ 1.42q software (http://rsb.info.nih.gov/ij).

### Statistical Analysis

In general, data are presented as arithmetic means ± SEM. Data were analyzed by a one-way ANOVA followed by a Holm-Sidak post-test, or a Kruskal-Wallis test followed by a Dunn’s test, as appropriate. A *p* value of <0.05 was considered significant. The software package GraphPad Prism 6.02 (GraphPad Software, San Diego, CA) was used for data analysis and preparation of graphs.

## Results

No adverse events occurred during these studies.

### Suppression of Inflammation and Remodelling in the Model of Mild Chronic Asthma

**Figure 2:**
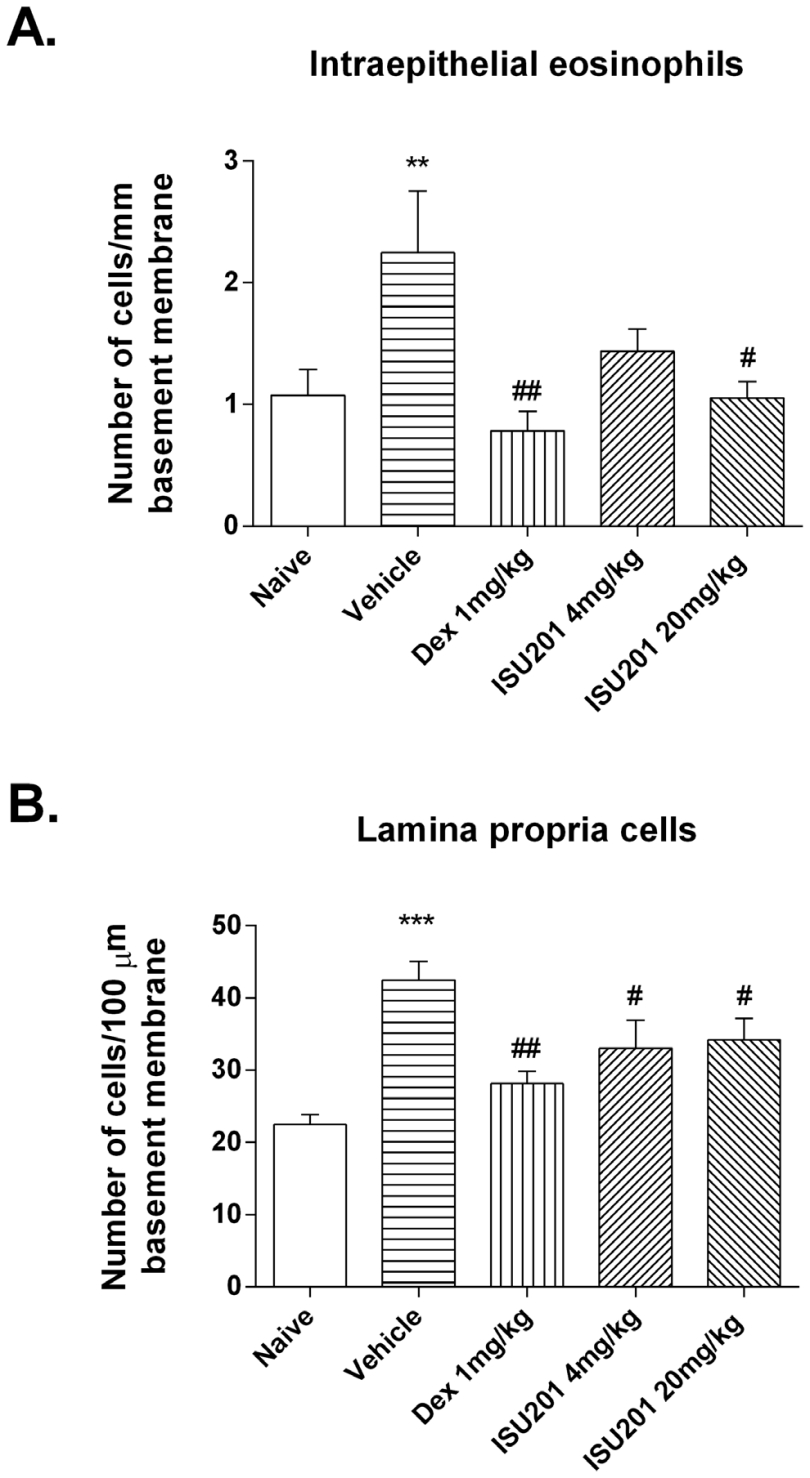
Airway inflammation in the model of mild chronic asthma. (A) Intraepithelial eosinophils (B) Inflammatory cells in the lamina propria of the trachea. OVA-exposed animals treated with vehicle alone are compared to unexposed animals or to animals treated with 4 or 20 mg/kg/day of ISU201, or of 1 mg/kg/day dexamethasone. Data are mean ± SEM (*n* = 6 samples per group). Significant differences relative to the naïve group are shown as **(p<0.01) and ***(p<0.001); relative to the vehicle-treated group are shown as #(p<0.05) and ##(p<0.01).

Treatment during the final two weeks of inhalational challenge with either dexamethasone or ISU201 suppressed the accumulation of both intraepithelial eosinophils (Fig. 2A) and chronic inflammatory cells, quantified as nuclear profiles in the lamina propria of the airways (Fig. 2B). The effects of ISU201 were at least partially dose-dependent, with 20 mg/kg of ISU201 suppressing eosinophil accumulation to an extent equivalent to 1 mg/kg of dexamethasone.

**Figure 3:**
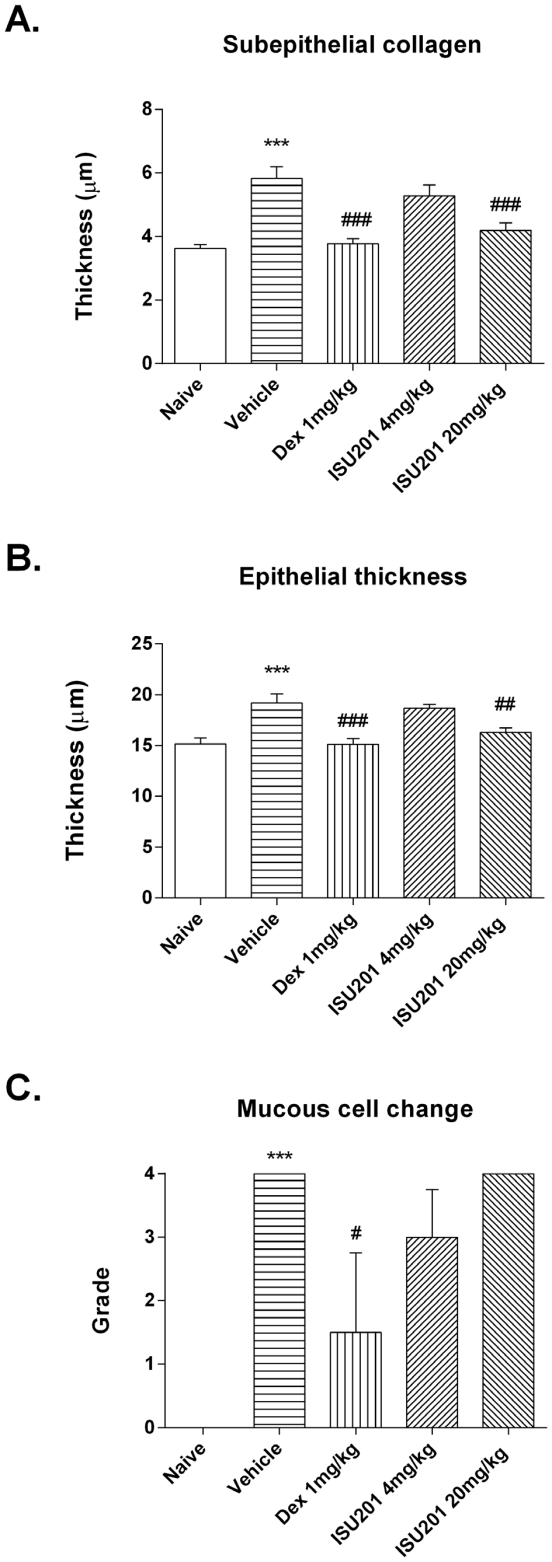
Airway remodelling in the model of mild chronic asthma. (A) Subepithelial accumulation of collagen (B) Epithelial thickening (C) Mucous cell change. OVA-exposed animals treated with vehicle alone are compared to unexposed animals or to animals treated with ISU201 or dexamethasone. Data are mean ± SEM (*n* = 6 samples per group) (A,B) or median ± interquartile range (C). Significant differences relative to the naïve group are shown as ***(p<0.001); relative to the vehicle-treated group are shown as #(p<0.05), ##(p<0.01) and ###(p<0.001).

Treatment during the final two weeks of inhalational challenge with dexamethasone or ISU201 also suppressed the development of subepithelial fibrosis (Fig. 3A) and of airway epithelial hypertrophy (Fig. 3B). Once again, the effects of ISU201 were dose-dependent, with relatively little effect at 4 mg/kg but an effect very similar to that of dexamethasone at 20 mg/kg of ISU201. In contrast, while treatment with dexamethasone suppressed the development of goblet cell hyperplasia/metaplasia in the epithelium of intrapulmonary airways, this was largely unaffected by ISU201 (Fig. 3C).

Underlying these effects was evidence of reduced expression of mRNA for a number of inflammatory cytokines in airway tissue. As shown in Table 1, both dexamethasone and ISU201 significantly suppressed the expression of IL-13, TNF-α and MIP-1α. Dexamethasone also inhibited the expression of additional cytokines, including IL-4, IL-17 and CXCL1.

**Table 1:** Effects of drug treatment on expression of cytokine mRNA in airway tissue in the chronic challenge model.

*Cytokine*	Vehicle	Dexamethasone	ISU201 4 mg/kg	ISU201 20 mg/kg
IL-4	57.6±6.1***	14.4±3.2^###^	66.4±6.7	64.8±9.1
IL-13	90.7±15.4***	12.5±2.9^###^	53.1±11.7^#^	51.8±9.3^#^
IL-17	43.6±11.3*	4.3±1.8^##^	41.5±10.8	27.8±9.5
TNF-α	5.2±1.3**	2.3±0.5^#^	2.1±0.6^#^	2.9±0.6
MIP-1α	9.5±1.1***	4.0±0.6^###^	7.5±1.0	5.0±0.8^##^
CXCL1	11.3±0.9***	3.4±0.6^###^	9.4±0.8	8.5±1.1

Values are fold expression relative to naïve animals, shown as mean ± SEM (*n* = 8). Significant differences compared to naïve animals are shown as *(p<0.05), **(p<0.01) and ***(p<0.001); compared to the vehicle-treated group as ^#^(p<0.05), ^##^(p<0.01) and ^###^(p<0.001).

### Suppression of Inflammation in the Model of an Acute Exacerbation of Asthma

**Figure 4:**
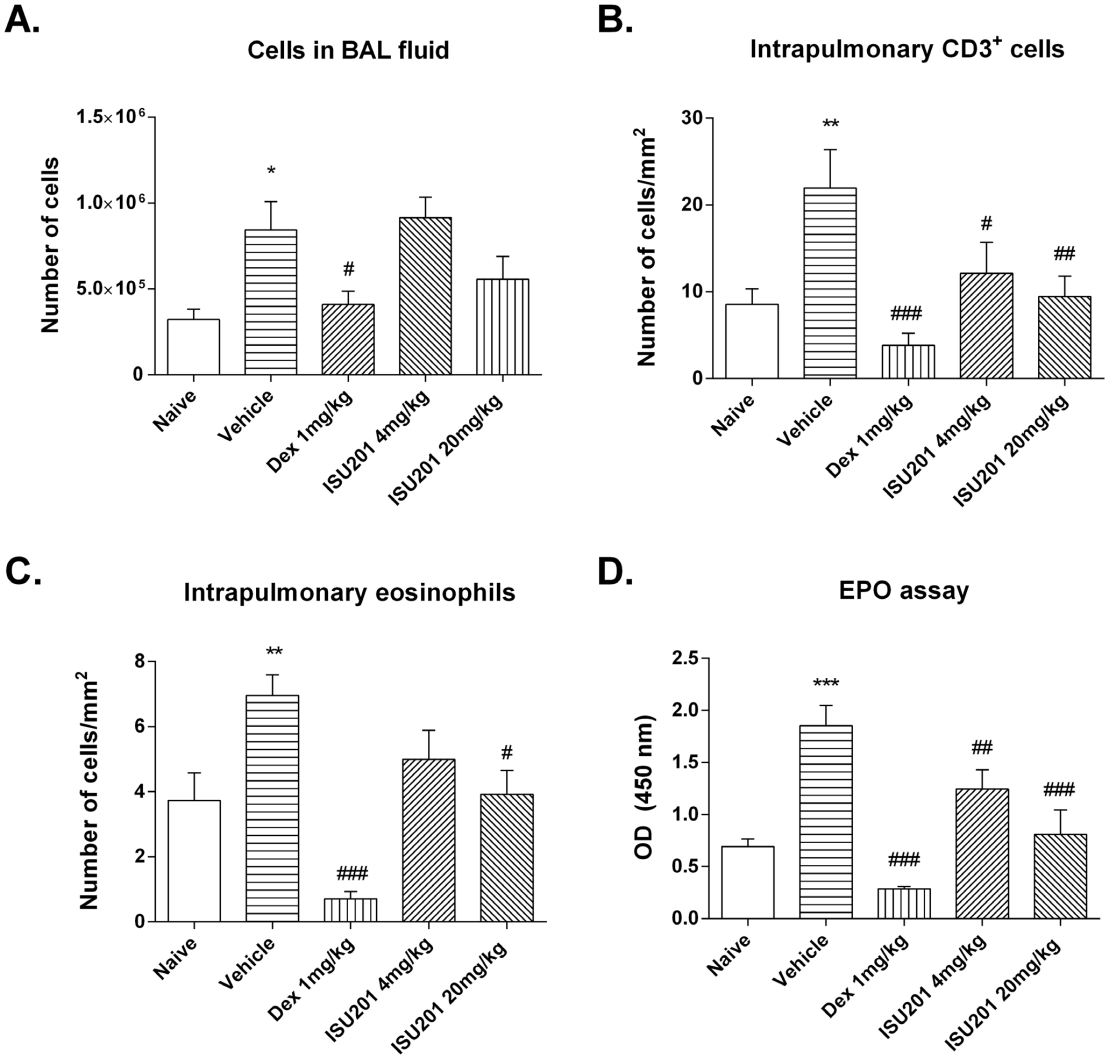
Airway inflammation in the model of an allergen-induced acute exacerbation of chronic asthma. (A) Numbers of cells in BAL fluid (B) Number of CD3^+^ cells in lung tissue, assessed by immunostaining (C) Number of eosinophils in lung tissue, assessed by cyanide-resistant peroxidase staining (D) Number of eosinophils in lung tissue, assessed by colorimetric assay for EPO. OVA-exposed animals treated with vehicle alone are compared to unexposed animals or to animals treated with ISU201 or dexamethasone. Data are mean ± SEM (*n* = 6 samples per group). Significant differences relative to the naïve group are shown as *(p<0.05), **(p<0.01) and ***(p<0.001); relative to the vehicle-treated group are shown as #(p<0.05), ##(p<0.01) and ###(p<0.001).

In this model, treatment with dexamethasone or ISU201 in the final 24 hours prior to the moderate-level inhalational challenge suppressed the increase in the total number of cells recovered by BAL. This effect was statistically significant for dexamethasone but not for ISU201, although the effect of 20 mg/kg was clearly greater than that of 4 mg/kg (Fig. 4A). In parallel, ISU201 at 20 mg/kg partially suppressed the increase in the percentage of lymphocytes and completely suppressed the increase in the percentage of neutrophils in BAL, the latter to an extent comparable to dexamethasone (not shown).

Both dexamethasone and ISU201 at 20 mg/kg significantly suppressed the accumulation of CD3^+^ T-lymphocytes and eosinophils in lung tissue (Fig. 4B, C). Suppression was much more marked in dexamethasone-treated mice, in which cell numbers were reduced below those in naïve animals. Suppression of eosinophil recruitment was confirmed by assessment of EPO activity in lung tissue homogenates (Fig. 4D).

Suppression of inflammation correlated with reduction in the concentrations of various Th2 and pro-inflammatory cytokine proteins in BAL fluid. In the case of ISU201, significant suppression was usually evident at a dose of 20 mg/kg, except for IL-6 and MIP-1β, which were also suppressed at 4 mg/kg (Table 2).

**Table 2:** Effects of drug treatment on cytokine concentrations in BAL fluid in the acute exacerbation model.

*Cytokine*	Naïve	Vehicle	Dexamethasone	ISU201 4 mg/kg	ISU201 20 mg/kg
IL-1β	6.8±1.6	13.9±1.3**	6.2±1.1^##^	12.9±2.3	6.9±1.2^##^
IL-4	1.3±0.1	11.5±2.2***	1.7±0.1^###^	12.4±2.4	3.7±0.6^##^
IL-5	0.9±0.3	2.0±0.2	0.8±0.4	2.3±0.6	0.6±0.1
IL-6	4.2±0.4	22.5±3.5***	3.9±0.6^###^	7.7±2.0^###^	5.5±1.1^###^
IL-10	0.9±0.2	2.5±0.5	0.9±0.2	3.2±1.0	1.4±0.6
IL-12p40	8.3±0.5	18.4±3.0***	3.9±0.9^###^	18.7±3.3	10.2±0.8^#^
IL-13	28.6±4.6	56.0±3.5	30.6±5.2	64.4±11.4	48.4±4.8
CXCL1	17.7±1.7	186.1±36.3*	83.8±7.4^#^	257.5±31.7	309.4±33.5
MIP-1α	2.7±0.0	20.1±5.6*	2.7±0.0^#^	17.5±6.5	7.4±4.8
MIP-1β	6.6±1.1	38.1±4.1***	9.2±1.9^###^	11.9±0.9^###^	13.5±2.2^###^
RANTES	1.7±0.1	8.5±1.5**	4.4±0.9^##^	9.7±1.5	5.6±0.5^#^

Values are pg/mL, shown as mean ± SEM (*n* = 8 animals per group). Significant differences compared to naïve animals are shown as *(p<0.05), **(p<0.01) and ***(p<0.001); compared to the vehicle-treated group as ^#^(p<0.05), ^##^(p<0.01) and ^###^(p<0.001).

### Suppression of Histone Acetylation in the Model of an Acute Exacerbation of Asthma

**Figure 5:**
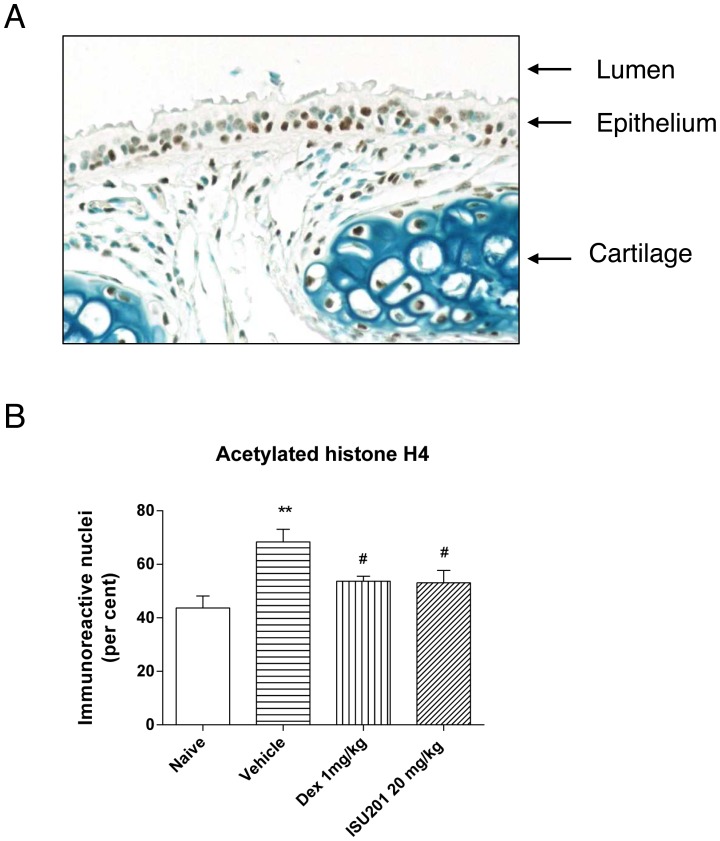
Immunoreactivity for acetylated histone H4 in nuclei of airway epithelial cells. (A) Photomicrograph of immunopositive nuclei (brown) in epithelial cells lining the trachea, original magnification ×400. (B) Percent positive cells in the trachea in the model of an allergen-induced acute exacerbation of chronic asthma. OVA-exposed animals treated with vehicle alone are compared to unexposed animals or to animals treated with ISU201 or dexamethasone. Data are mean ± SEM (*n* = 6 samples per group). Significant differences relative to the naïve group are shown as **(p<0.01); relative to the vehicle-treated group are shown as #(p<0.05).

Following induction of an experimental acute exacerbation of chronic asthma, there was a marked increase in the proportion of airway epithelial cells that exhibited nuclear immunoreactivity for acetylated histone H4. This increase was significantly reduced in animals that had been treated with either dexamethasone or ISU201 at 20 mg/kg, with comparable effects for both drugs (Fig. 5). Induction of an acute exacerbation was not associated with an increase in immunoreactivity for acetylated histone H3, nor was there any effect of drug treatment (not shown).

### Cellular Effects in the Model of an Acute Exacerbation of Asthma

Treatment with ISU201 at 24 and 2 hours prior to induction of an experimental acute exacerbation was very effective in suppressing the subsequent production of cytokines by isolated PBLN cells restimulated in vitro. Even at 4 mg/kg, for most cytokines tested ISU201 reduced levels in culture supernatants to at least the same extent as dexamethasone at 1 mg/kg (Table 3).

**Table 3:** Effects of drug treatment on cytokine concentrations in supernatants of restimulated PBLN cells from the acute exacerbation model.

*Cytokine*	Naïve	Vehicle	Dexamethasone	ISU201 4 mg/kg
IL-4	3.2±1.3	909.5±174.7**	308.8±147.2^#^	179.6±97.1^##^
IL-6	15.2±2.4	82.2±19.6**	82.9±5.5	27.5±7.4^#^
IL-10	5.3±1.7	424.1±66.7***	122.2±28.2^##^	135.3±62.1^##^
IL-13	35.1±1.0	254.7±74.0*	38.7±1.7^#^	337±1.3
MIP-1β	13.7±6.1	492.3±81.9***	215.5±16.8^#^	141.1±58.2^##^

Values are pg/mL, shown as mean ± SEM (*n* = 3 samples per group, pooled pairs from 6 animals). Significant differences compared to naïve animals are shown as *(p<0.05), **(p<0.01) and ***(p<0.001); compared to the vehicle-treated group as ^#^(p<0.05) and ^##^(p<0.01). For technical reasons, sufficient numbers of cells could not be collected from animals treated with 20 mg/kg of ISU201.

To assess whether the observed reduction was a result of the drug treatment having affected the phenotype of the cells that were being assessed for restimulation in culture, surface markers expressed by PBLN cells were assessed by flow cytometry. Compared to naïve mice (59.3±0.2%), the relative proportion of CD3+CD4+ cells did not change significantly in PBLN cells from animals in which an acute exacerbation was induced (58.4±0.2%), or in animals treated with dexamethasone (62.0±0.2%). In animals treated with ISU201, there was a small decrease in the proportion of CD3+CD4+ cells (49.7±0.2%) with a corresponding increase in CD3-CD19+ B cells. In all groups, total CD3+ cells comprised 60–80% of cells in the lymphocyte gate. Thus the effects of ISU201 on production of cytokines appeared to be the result of a direct or indirect effect on the responses of T cells, rather than on their number.

In animals treated with ISU201, expression of mRNA for a variety of pro-inflammatory cytokines by AM was also suppressed. While this effect was usually less marked than that observed following pre-treatment with dexamethasone, it was nevertheless significant for nearly all of the cytokines assessed, and at least in part was dose-dependent (Table 4).

**Table 4:** Effects of drug treatment on expression of cytokine mRNA by AM from the acute exacerbation model.

*Cytokine*	Vehicle	Dexamethasone	ISU201 4 mg/kg	ISU201 20 mg/kg
IL-1β	64.1±11.4***	5.5±3.6^###^	46.9±3.5	28.2±2.4^##^
IL-6	6.6±1.1***	1.0±0.6^###^	4.3±1.1	5.5±0.7
TNF-α	9.1±0.7***	2.3±0.6^###^	7.2±0.6	5.7±1.1^#^
CXCL1	10.2±1.2***	7.4±1.2	7.6±1.1	5.9±0.4^##^
CCL24	54.5±6.7***	3.6±1.4^###^	23.4±3.9^###^	20.5±6.3^###^

Values are fold expression relative to naïve animals, shown as mean ± SEM (*n* = 6). Significant differences compared to naïve animals are shown as ***(p<0.001); compared to the vehicle-treated group as ^#^(p<0.05), ^##^(p<0.01) and ^###^(p<0.001).

### Activity of ISU201 in vitro

#### Lymphocytes

To specifically assess whether there was a direct effect of ISU201 on lymphocytes, PBLN cells were collected from untreated animals in which an acute exacerbation had been induced, then restimulated with OVA in vitro, and the effects of drug treatment in vitro on the cytokine response were examined. The results (Table 5) convincingly demonstrated that ISU201 directly suppressed cytokine production by lymphocytes in vitro, in a concentration-dependent manner.

**Table 5:** Effects of drug treatment in vitro on cytokine concentrations in supernatants of restimulated PBLN cells.

*Cytokine*	Medium	OVA	OVA+ISU201 1 µg/mL	OVA+ISU201 3 µg/mL	OVA+ISU201 10 µg/mL
IL-4	6.0±1.1	211.5±61.8**	198.4±40.3	110.6±19.2	76.9±24
IL-6	30.5±5.1	49.8±2.7**	48.6±2.9	38.2±5.2	26.4±2.9^##^
IL-10	28.9±4.5	64.3±1.5**	77.6±4.8	53.4±10.1	38.2±6.0^#^
IL-12p40	18.6±2.2	26.0±2.8	20.8±3.2	17.0±4.4	8.8±1.8^##^
IL-13	56.9±17.6	133.8±16.8*	143.0±24.7	91.7±14.6	41.4±6.5^##^
IL-17	6.6±1.4	21.4±3.9	23.4±3.4	8.1±2.0	4.7±2.3^#^
MIP-1β	18.6±2.4	55.4±2.3***	68.6±9.1	50.6±5.4	27.3±8.3^##^
RANTES	15.6±1.1	34.4±3.3**	25.1±2.8	18.7±2.0^##^	20.2±3.7^#^

Values are pg/mL, shown as mean ± SEM (*n* = 4 samples per group, pooled pairs from 8 animals). Significant differences relative to cells cultured in medium only are shown as * (p<0.05), ** (p<0.01) and *** (p<0.001); relative to cells restimulated with OVA are shown as ^#^(p<0.05) and ^##^(p<0.01).

#### Macrophages

To specifically assess whether there was a direct effect of ISU201 on AM, these cells were collected from animals in which an acute exacerbation had been induced, and expression of cytokine mRNA by cells treated with ISU201 for 4 hours in vitro was assessed. This preliminary experiment suggested that ISU201 directly suppressed the expression of TNF-α, but had little effect on other pro-inflammatory cytokines (not shown). Because the limited availability of AM recovered by lavage restricted further investigation, in subsequent experiments the MH-S line of differentiated AM was employed. We have previously shown that these cells can activated by IL-33, resulting in a profile of expression of pro-inflammatory cytokines similar to that observed in AM isolated by lavage from the acute exacerbation model [28]. Compared to dexamethasone, which markedly decreased the expression of several relevant cytokines, we found that in these cells the effects of ISU201 were more restricted, and again of the cytokines assessed only TNF-α was significantly suppressed (Table 6).

**Table 6:** Effects of drug treatment in-S cells.

*Cytokine*	IL-33	IL-33+ Dexamethasone	IL-33+ ISU201 10 µg/mL
IL-1β	119.2±6.5***	0.9±0.1^###^	101.2±7.7
IL-6	100.6±8.5***	3.6±0.2^###^	139.6±9.5
TNF-α	4.4±0.3***	0.4±0.0^###^	2.7±0.8^#^

Values are fold expression relative to medium alone, shown as mean ± SEM (*n* = 6). Significant differences compared to cells cultured in medium alone are shown as *** (p<0.001), compared to IL-33-treated cells as ^#^(p<0.05) and ^###^(p<0.001).

**Figure 6:**
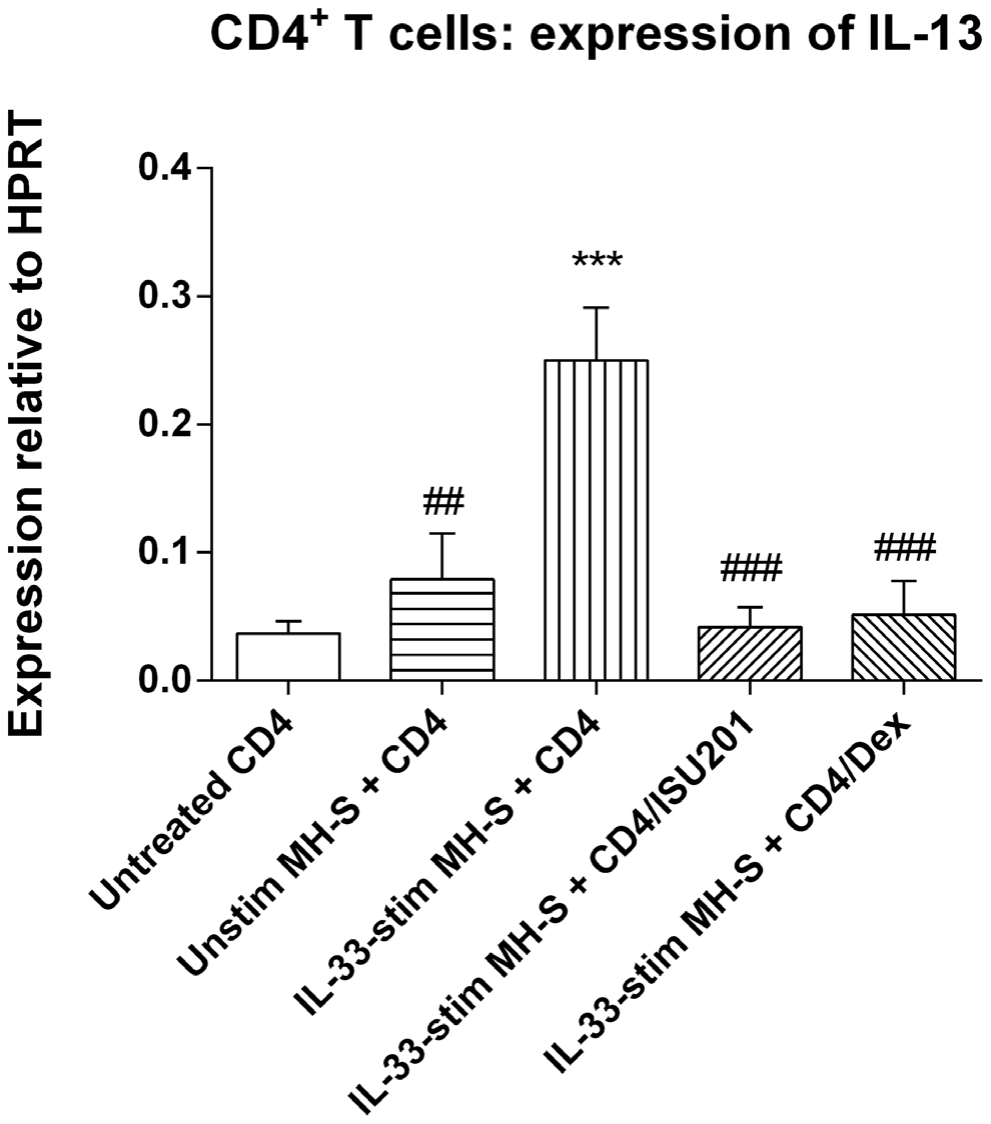
Relative expression of mRNA for IL-13 by primed CD4^+^ T cells co-cultured with MH-S cells. Cells were either untreated or stimulated with IL-33 in the absence or presence of ISU201 or dexamethasone. Data are mean ± SEM (*n* = 3 samples per group). Significant differences relative to unstimulated cells are shown as *** (p<0.001); relative to IL-33-stimulated cells in medium alone are shown as ##(p<0.01) and ###(p<0.001).

Subsequently, MH-S cells were used to assess the capacity of ISU201 to suppress macrophage function with respect to activation of primed CD4+ T cells to secrete cytokines. PBLN cells were obtained from systemically sensitised mice after 4 weeks of chronic challenge with low levels of aerosolised OVA, and CD4+ T cells were isolated. These cells were then co-cultured for 4 hours with adherent MH-S cells that had been activated with IL-33 and pre-treated with either ISU201 (10 µg/mL) or dexamethasone (40 µg/mL). After this, the non-adherent CD4+ cells were recovered, mRNA was extracted and induction of Th2 cytokine expression was assessed. Treatment with ISU201 suppressed the upregulation of mRNA for IL-13 to a degree comparable to that in response to treatment with dexamethasone (Fig. 6). However, IL-4 and IL-5 were not induced by MH-S cells in this co-culture system, in contrast to our findings using AM obtained by lavage [17].

#### Airway epithelial cells

**Figure 7:**
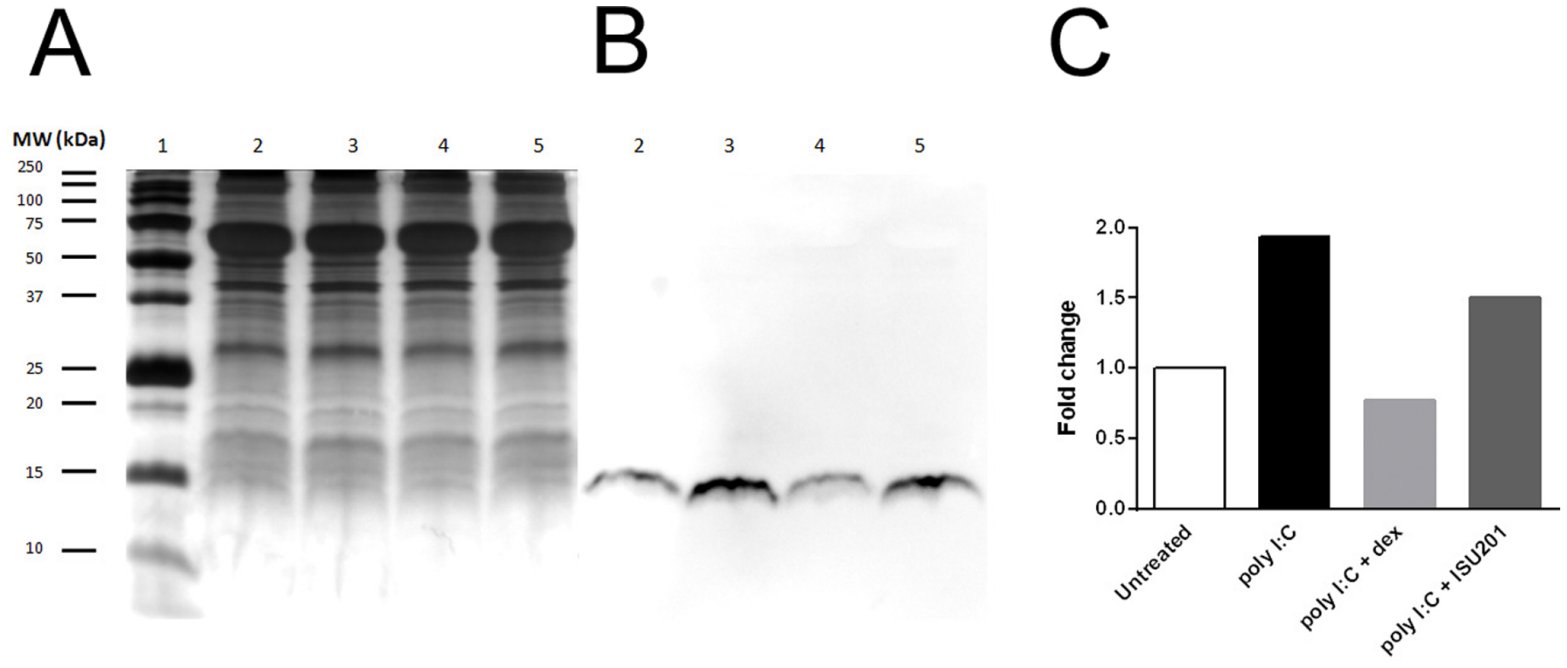
Effects of drug treatment in vitro on acetylation of histone H4. AEC were stimulated with poly I:C and treated with either dexamethasone or ISU201. (A) Silver-stained gel (B) Western blot, chemiluminescent detection for 60 sec (C) Relative expression by densitometry. Lane 1 =  molecular weight markers, lane 2 =  unstimulated cells, lane 3 =  cells stimulated with poly I:C, lane 4 =  cells stimulated with poly I:C and treated with dexamethasone, lane 5 =  cells stimulated with poly I:C and treated with ISU201. Results are representative of 3 separate experiments.

The increase in acetylation of histone H4 in AEC stimulated in vitro with poly I:C was reduced in cells that had been treated with either dexamethasone or ISU201, although the effect appeared to be greater for dexamethasone (Fig. 7).

## Discussion

In previous studies by Isu Abxis, the ECD protein component of BST2 and the stabilised drug form now referred to as ISU201 were shown to have anti-inflammatory activity, including in a short-term model of allergic inflammation of the airways ([18] and Yoo et al, manuscript in preparation). In the present study, we provide evidence of the capacity of ISU201 to inhibit airway inflammation and remodelling in models of mild chronic asthma and an acute exacerbation of asthma, which reproduce many of the features of the clinical disease. In the chronic challenge model, these included eosinophil recruitment into the epithelial layer of the conducting airways, chronic inflammation in the airway wall with accumulation of CD4+ T-lymphocytes, and changes of remodelling such as sub-epithelial fibrosis, epithelial hypertrophy and goblet cell metaplasia. ISU201 was as effective as dexamethasone with respect to suppression of airway inflammation and most changes of remodelling, the notable exception being a lack of effect on goblet cell hyperplasia/metaplasia. Two weeks of treatment could have induced production of antibodies to the human protein component of ISU201, but if so, this clearly had little impact on its biological activity. Similarly, ISU201 was effective in the model of an allergen-induced acute exacerbation of chronic asthma, although its activity was less marked than that of dexamethasone.

We also provide evidence that in the model of an acute exacerbation of asthma, ISU201 acts on multiple cellular targets, suppressing the production of a variety of pro-inflammatory cytokines by lymphocytes and macrophages, as well as inhibiting the functional interaction between these cells. This broad spectrum of anti-inflammatory activity of ISU201 is of considerable interest, as it suggests that the effects of this protein are in many respects similar to those of glucocorticosteroids. The suppressive effects on multiple key pro-inflammatory mediators and Th2 cytokines, as well as on macrophage function, are in contrast to novel therapeutic interventions directed against a single mediator or mediators of a single class, such as monoclonal antibodies against individual cytokines or shared receptors.

In vitro cellular studies indicated that ISU201 inhibited production of a range of cytokines by lymphocytes. However, whereas the drug markedly inhibited cytokine expression by macrophages recovered from animals treated in vivo, the effects of ISU201 on activated macrophages in culture were limited to suppression of TNF-α. This finding suggests that at least some of the inhibitory effect of the drug on macrophages is indirect, possibly via its effect on lymphocytes.

Relatively little is known about the potential of BST2 or its ECD to modulate inflammatory responses. BST2, which is also known as CD317 or tetherin, is ubiquitously expressed by type I interferon-producing cells and is thought to play an important role in the innate host response to enveloped viruses, by inhibiting their spread [29], [30]. Nevertheless, studies in BST2-deficient mice imply a more complex role for this protein in regulating viral infection [31]. In addition, BST2 has been suggested to be an endogenous ligand for LILRA4, also known as ILT7, and to be able to inhibit cytokine production by cells expressing this immunoregulatory receptor [32], although its biological role remains contentious [33].

On the basis of studies in vitro using an airway epithelial cell line, Yoo et al have suggested that at a cellular level, the anti-inflammatory effects of ISU201 might be related to its capacity to inhibit phosphorylation of NF-κB p65 (manuscript in preparation). Nuclear translocation of NF-κB in the airway epithelium is increased in asthma [34] and may be an early event in the response to allergen challenge [35]. In the present investigation, we provide evidence that at a tissue level, these effects on NF-κB may be related to the ability of ISU201 to suppress the acetylation of histones. Activation of inflammatory genes is well recognised to be associated with histone acetylation, and the anti-inflammatory activity of glucocorticosteroids is substantially related to their capacity to recruit histone deacetylases to the nucleus and reverse this process [36]. Acetylation of histone H4 is particularly associated with inflammatory diseases, and has been demonstrated in mucosal tissues in both chronic obstructive pulmonary disease [37] and inflammatory bowel disease [38]. Thus our finding that suppression of inflammation correlated with evidence of significantly reduced acetylation of histone H4 in airway epithelial cells of animals treated with ISU201, both in vivo and in vitro, provides at least one potential mechanism of action of this compound.

In conclusion, we have demonstrated that the novel drug ISU201 is a broad-spectrum inhibitor of both airway inflammation and remodelling, in models of mild chronic asthma and an acute exacerbation of asthma. In many respects, its range of effects resembles that of the glucocorticoid dexamethasone and is in contrast to drugs which target specific mediators. While any clinical application will need to take into account that asthma is a syndrome, with several distinct phenotypes now recognised [39], [40] which differ in terms of response to therapy, ISU201 could potentially be an alternative or an adjunct to glucocorticoids for the treatment of asthma.
